# Artificial intelligence in anesthesiology: a bibliometric analysis

**DOI:** 10.1186/s13741-024-00480-x

**Published:** 2024-12-23

**Authors:** Bi-Hua Xie, Ting-Ting Li, Feng-Ting Ma, Qi-Jun Li, Qiu-Xia Xiao, Liu-Lin Xiong, Fei Liu

**Affiliations:** 1 Department of Anesthesiology, West China Hospital, Sichuan University, Chengdu, 610041 Sichuan China; 2 Department of Anesthesiology, The Third People’s Hospital of Yibin, Yibin, 644000 Sichuan China; 3 Department of Anesthesiology, The First People’s Hospital of Shuangliu District, Chengdu, 610041 Sichuan China; 4 School of Pharmacy, Zunyi Medical University, Zunyi, 563000 Guizhou China; 5 Department of Anesthesiology, The Third Affiliated Hospital of Zunyi Medical University (The First People’s Hospital of Zunyi), Zunyi, 563000 Guizhou China

**Keywords:** Artificial intelligence, Anesthesiology, Ultrasound-guided regional anesthesia, Postoperative pain, Airway management, Predict, Depth of anesthesia, Closed-loop infusion

## Abstract

The application of artificial intelligence (AI) in anesthesiology has become increasingly widespread. However, no previous study has analyzed this field from the bibliometric analysis dimension. The objective of this paper was to assess the global research trends in AI in anesthesiology using bibliometric software. Literatures relevant to AI and anesthesiology were retrieved from the Web of Science until 10 April 2024 and were visualized and analyzed using Excel, CiteSpace, and VOSviewer. After screening, 491 studies were included in the final bibliometric analysis. The growth rate of publications, countries, institutions, authors, journals, literature co-citations, and keyword co-occurrences was computed. The number of publications increased annually since 2018, with the most significant contributions from the USA, China, and England. The top 3 institutions were Yuan Ze University, National Taiwan University, and Brunel University London. The top three journals were *Anesthesia & Analgesia*, *BMC Anesthesiology*, and *the British Journal of Anaesthesia*. The researches on the application of AI in predicting hypotension have been extensive and represented a hotspot and frontier. In terms of keyword co-occurrence cluster analysis, keywords were categorized into four clusters: ultrasound-guided regional anesthesia, postoperative pain and airway management, prediction, depth of anesthesia (DoA), and intraoperative drug infusion. This analysis provides a systematic analysis on the literature regarding the AI-related research in the field of anesthesiology, which may help researchers and anesthesiologists better understand the research trend of anesthesia-related AI.

**Supplementary Information:**

The online version contains supplementary material available at 10.1186/s13741-024-00480-x.

## Introduction

In the 1980s, medical malpractice insurance premiums for the Department of Anesthesia at Harvard Medical School increased rapidly. After an investigation, it was found that most of these could be avoided with continuous monitoring. Consequently, standard monitoring guidelines for anesthesia were proposed (Eichhorn, et al. 1986). These guidelines were subsequently recognized and promoted by the American Society of Anesthesiologists and were quickly applied in various countries (Epstein 1987). Modern anesthesia relies heavily on a large amount of monitoring data to manage patients and make decisions rapidly in a short time, which largely depends on the experience of anesthesiologists (Connor 2019). With the advent of perioperative medicine and precision medicine, anesthesiologists are required to process more data and expend more energy. If data processing becomes concise and fast, it can benefit both patients and anesthesiologists. The concept of AI was first introduced in a meeting in 1956. With the development of AI, data processing has become simpler. For instance, the bispectral index, which converted complex electroencephalography (EEG), signals into a numerical value ranging from 0 to 100 (Rampil 1998), enabling anesthesiologists to make quicker judgments on the DoA. Various predictive models had been developed, such as those for predicting difficult airways, hypotension, and postoperative mortality (Hayasaka et al. 2021; Lee, et al. 2020; Fritz et al. 2019). Driven by AI, multiple data sources have been used to provide personalized treatment plans for patients (Lonsdale et al. 2022). Rodovan Richta and Masse Bloomfield summarized the three stages of the development of anesthesiology: the first stage involves “tools,” such as laryngoscopes and tracheal tubes; the second stage involves “machines,” such as infusion pumps and anesthesia workstations; and the third stage involves “automation,” when machines controlled by humans will be replaced by automatic algorithms (Moon and Cannesson 2022). In summary, the study of AI in anesthesiology has become increasingly widespread.

Bibliometrics is a method that is used to conduct both qualitative and quantitative reviews and analyses of research within a particular field (Ninkov et al. 2022), providing an objective view of hotpots and frontiers. Although the literatures related to AI in anesthesiology grow rapidly, a comprehensive bibliometric analysis of these literatures is still lacking. The aim of this study was to analyze the current research status and development trends of AI in anesthesiology and identify the hotspots and frontiers in this field.

## Methods

### Data source and search strategy

**Fig. 1:**
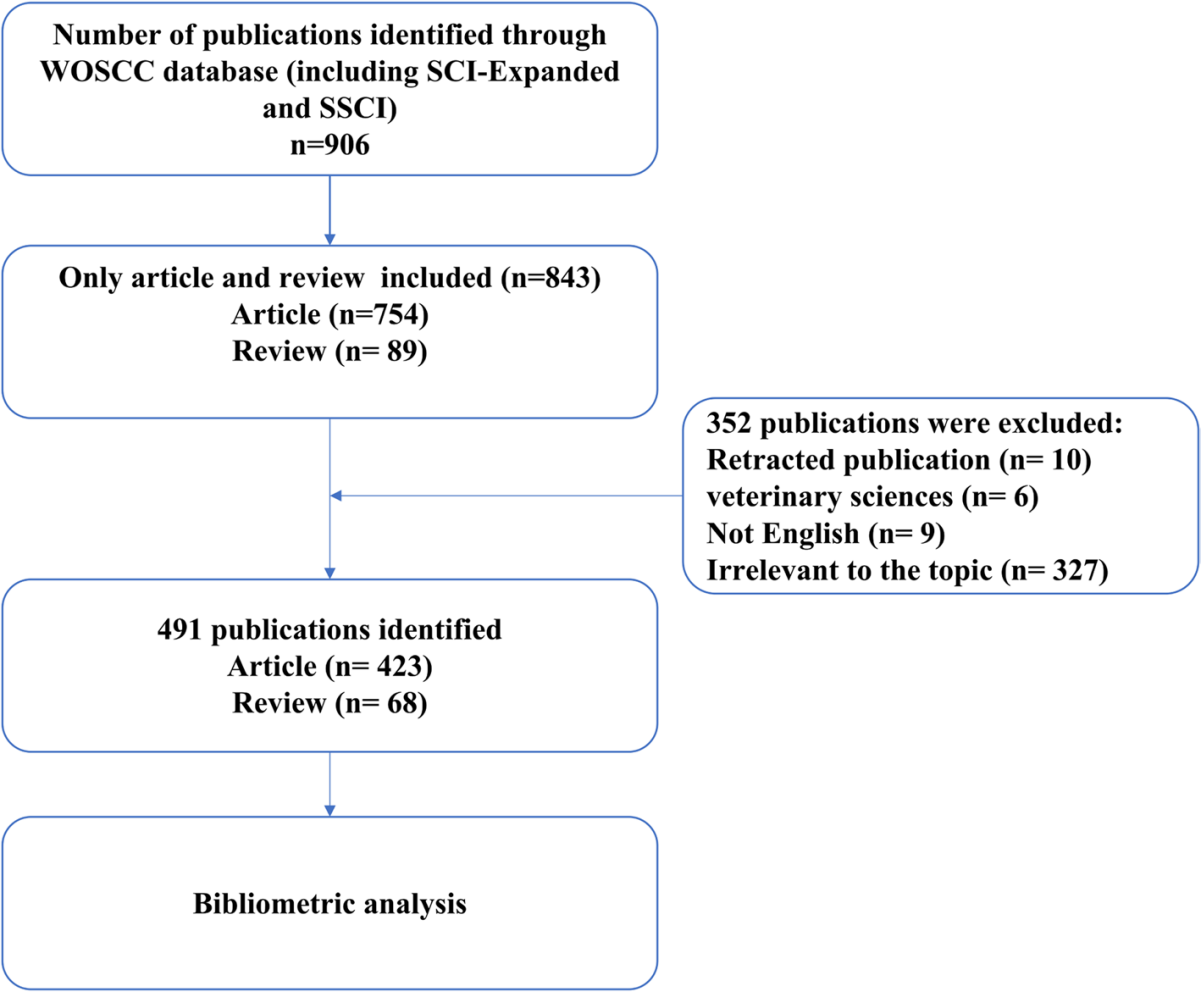
Flow chart of literature screening related to AI in anesthesiology

The Web of Science is an authoritative academic database that has been widely used by researchers around the world. The literature data was obtained from the Web of Science Core Collection database, which includes the Science Citation Index Expanded and Social Sciences Citation Index. The search strategy was TS = ((“artificial intelligence” OR “machine learning” OR “deep learning” OR “neural network” OR “convolutional neural network” OR “fuzzy logic” OR “reinforcement learning” OR “supervised learning” OR “unsupervised learning” OR “natural language processing” OR “computer vision” OR “big data” OR “computerized analysis”) AND (“anesthes*”)). The search was conducted up to April 2024, and only articles and reviews were included. The flow chart of the literature screening process is illustrated in Fig. 1.

### Data collection

The literature was independently screened by two authors (B. H. X. and T. T. L.), and studies not related to the search strategy were excluded. Discussions were held with a third author (F. T. M.) to resolve any disagreements. Data, including titles, keywords, authors, countries, journals, institutions, and references, were extracted from the Web of Science and were ultimately included in this study.

### The bibliometric analysis method

Based on the screened literature, an analysis of the number of publications on AI in anesthesiology was visualized using Microsoft Excel 2016. Then, bibliometric analysis was performed using CiteSpace 6.3. R 1 and VOSviewer 1.6.20 were used to analyze the distribution of countries, institutions, authors and journals, the co-citation of literature, and the clustering of keyword co-occurrence. The most recent impact factors were obtained from the most recent edition of the Journal Citation Report.

## Results

The 491 studies included in this study were from 48 countries, 715 institutions, and 2219 authors. They were published in 218 journals and cited 16,929 references from 5070 journals.

### Annual number of publications and cumulative number of publications

**Fig. 2:**
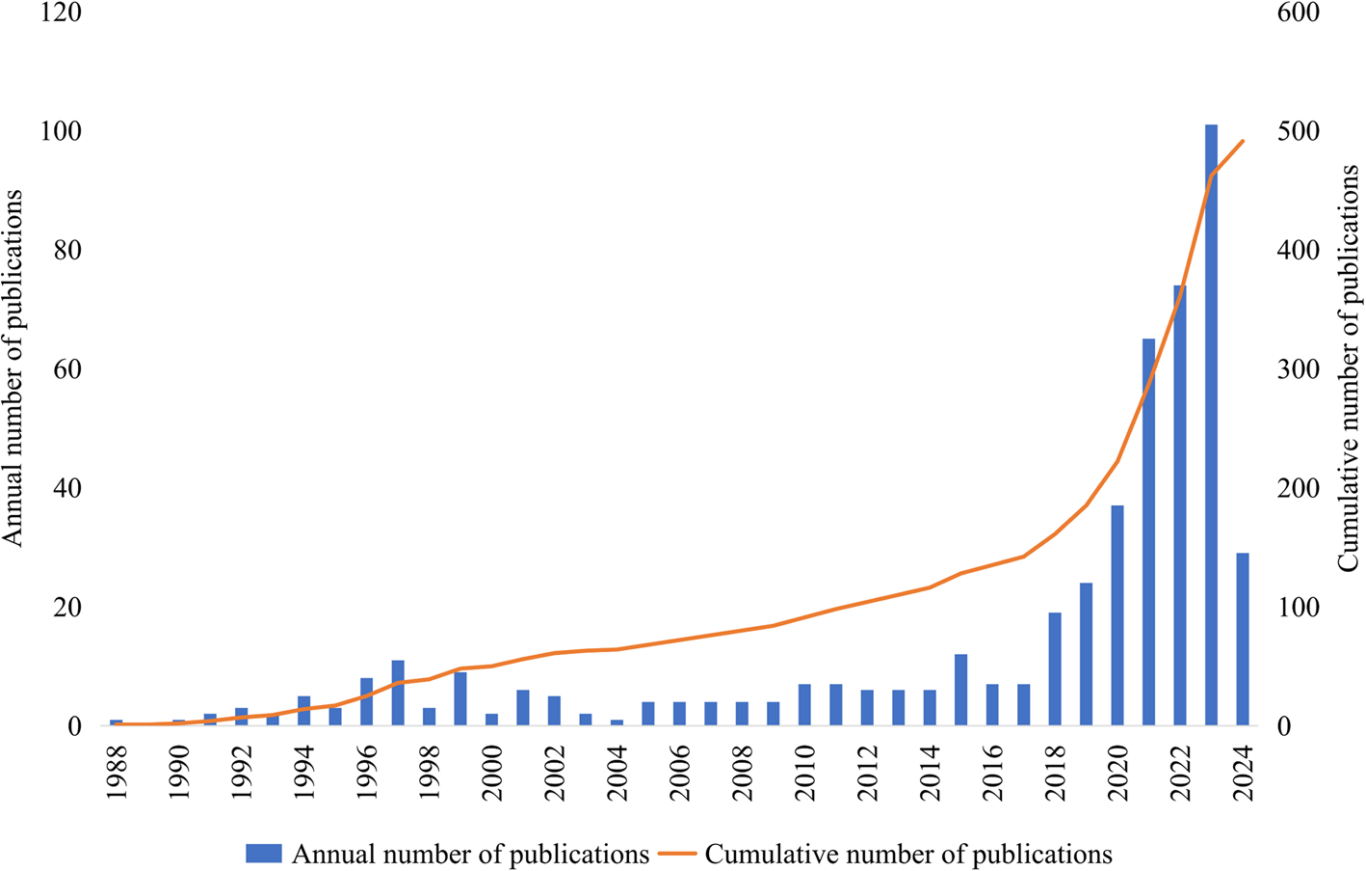
Annual and cumulative number of publications published on AI and anesthesiology

Figure 2 shows the annual and cumulative number of publications related to AI in anesthesiology from 1986 to 2024. In general, the number of publications was low and stable before 2018. After 2018, the number of publications increased annually, with 19 studies published in 2018 and 101 in 2023. The cumulative number of publications increased gradually, with an exponential growth trend, especially after 2018.

### Distribution of countries, institutions, authors, and journals

**Fig. 3:**
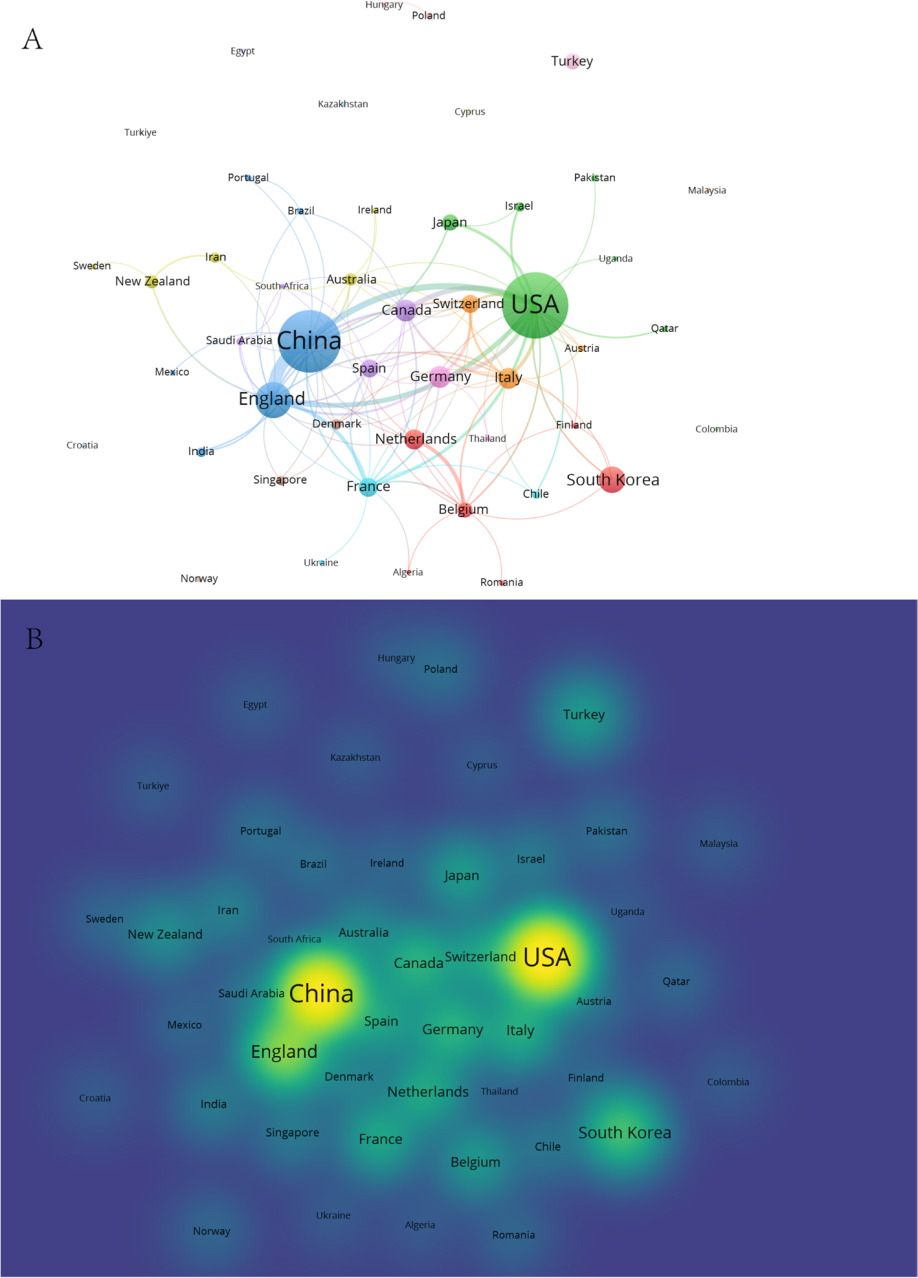
The distribution of countries in terms of AI and anesthesiology. **A** Countries with more than one publication. Each node represents a country, and the size of the node indicates the number of publications. The connections between nodes indicate collaborations between countries, with the thickness of the lines representing the strength of the collaboration. **B** A density visualization of countries

A total of 48 countries published research on AI and anesthesiology (Fig. 3). The top 10 countries in terms of the number of publications were the USA, China, England, South Korea, Canada, Germany, Italy, the Netherlands, France, and Spain. The strongest collaborations between countries were between the USA, China, and England.

**Fig. 4:**
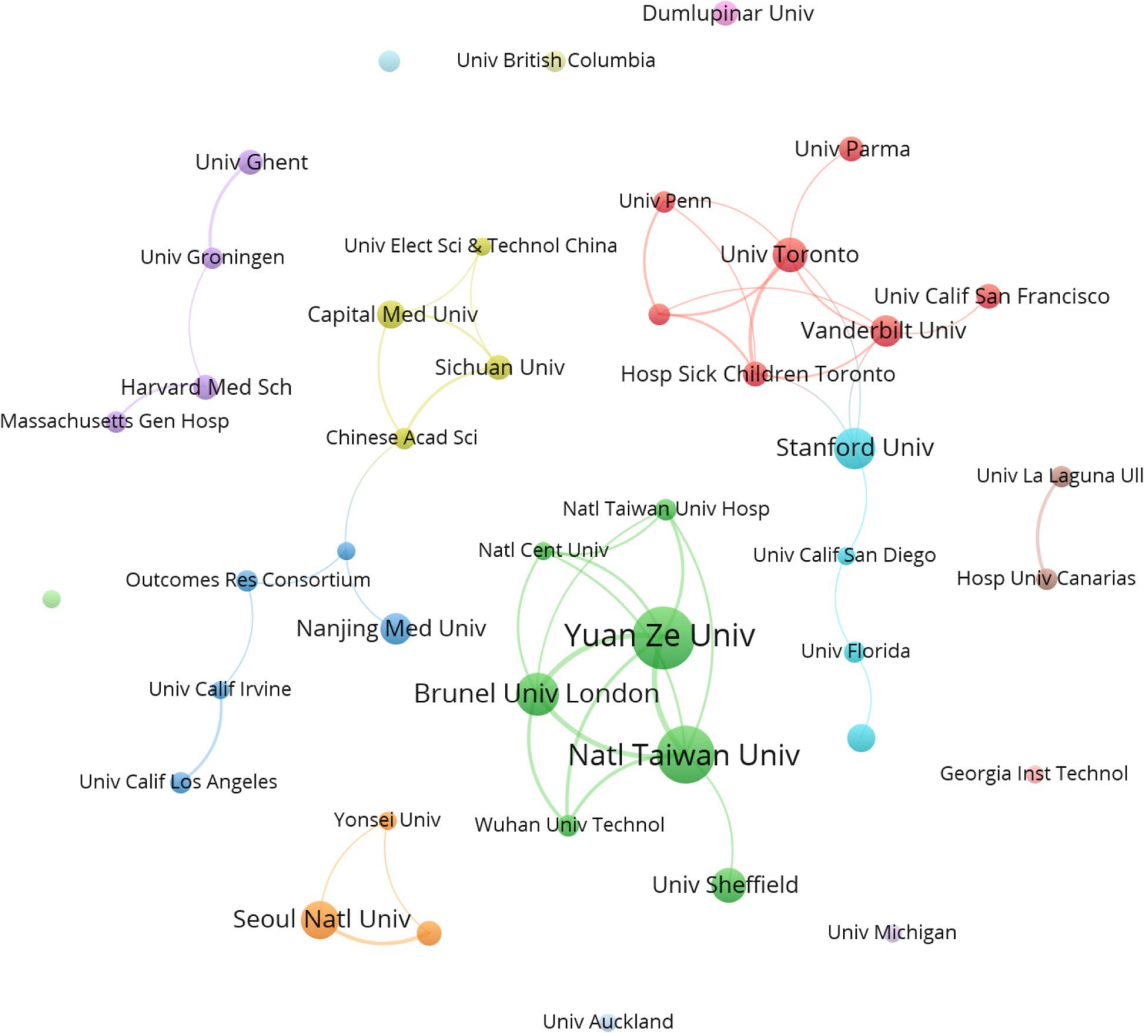
Distribution of institutions in AI and anesthesiology. Institutions with more than five publications. Each node represents an institution, with the size of the node indicating the number of publications. Connections between nodes indicate collaborations between institutions, and the thickness of the lines represents the strength of the collaboration

A total of 715 institutions published research on AI and anesthesiology, among which 43 institutions published more than 5 papers (Fig. 4). The institution with the most publications was Yuan Ze University (20), followed by National Taiwan University (18) and Brunel University London (13) (Supplementary Table 1). Both of the first two universities were located in Taiwan, China.

The top 5 authors in AI and anesthesiology in terms of the number of publications were Shieh, Jiann-Shing (18); Fan, Shou-Zen (16); Linkens, D. A. (13); Abbod, Maysam F. (13); and Lee, Hyung-Chul (8) (Supplementary Table 2).

**Fig. 5:**
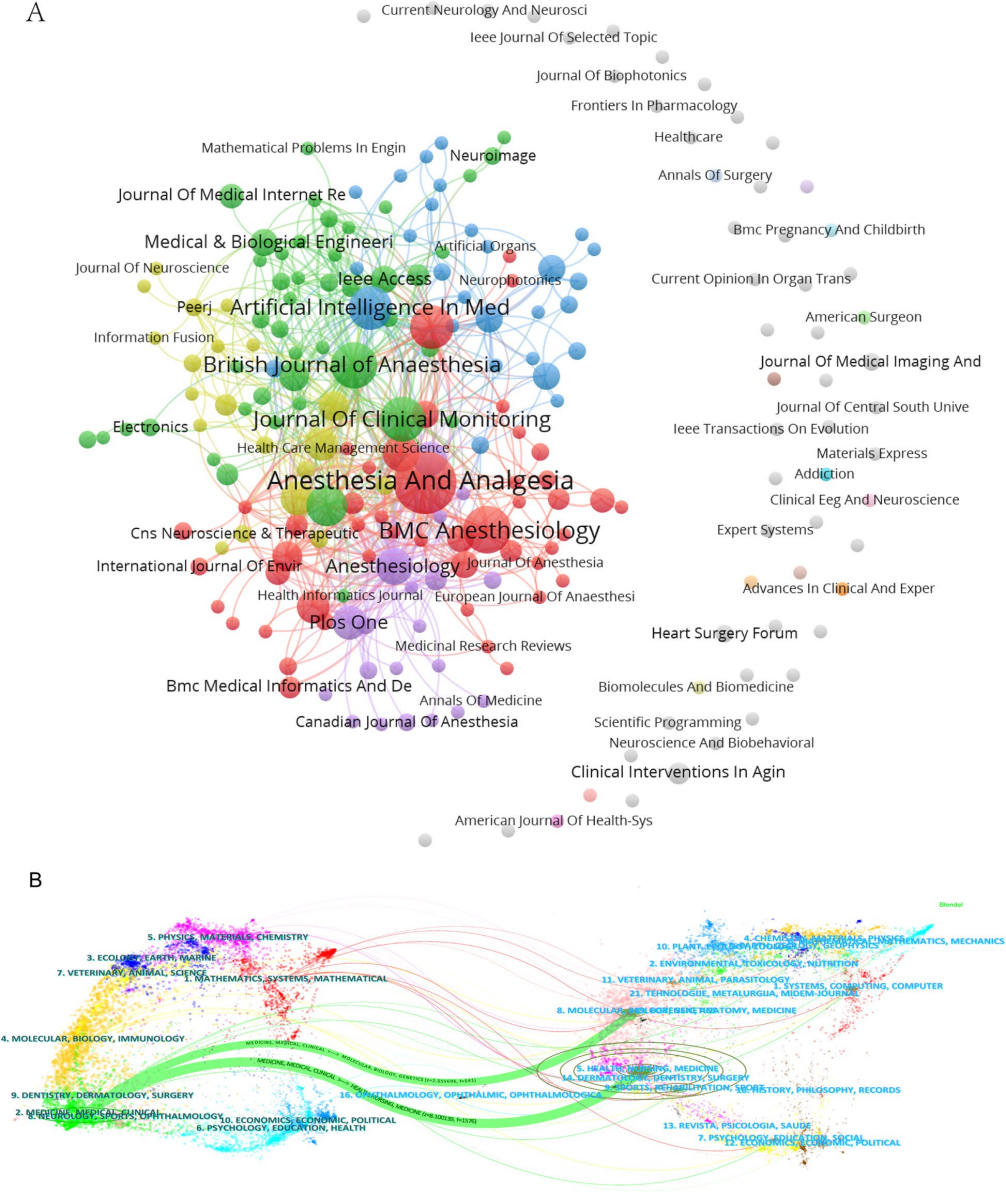
Distribution of journals. **A** Two-hundred eighteen journals on artificial intelligence in anesthesiology. **B** Dual-map overlays of the journals. The left side represents citing journals, symbolizing the frontier of knowledge; the right side represents cited journals, symbolizing the foundation of knowledge; and the lines represent citation connections

Figure 5A displays 218 journals that published papers in AI and anesthesiology. Figure 5B uses dual-map overlays created by CiteSpace to show the thematic distribution, citation trajectory, and research center shift of the journals. The map showed that the main themes of the cited journals were *molecular*, *biology*, *genetics*, *health*, *nursing*, and *medicine*. The main themes of the citing journals were *medicine*, *medical*, and *clinical*. From the journal themes, it could be seen that the research field was transforming from basic to clinical, which was precisely the ultimate goal of the application of AI in anesthesiology.

Table 1 shows the top 10 journals in AI and anesthesiology in terms of the number of publications and the total and average citations. The top three journals were *Anesthesia & Analgesia*, *BMC Anesthesiology*, and *British Journal of Anaesthesia*.

**Table 1:** The top 10 journals in AI and anesthesiology in terms of the number of publications

Rank	Journal	Publications	Total citations	Average citations	IF JCR2023
1	*Anesthesia & Analgesia*	30	429	14.3	4.6
2	*BMC Anesthesiology*	17	73	4.29	2.3
3	*British Journal of Anaesthesia*	16	484	30.25	9.1
4	*Journal of Clinical Monitoring and Computing*	15	161	10.73	2.0
5	*Artificial Intelligence in Medicine*	14	420	30	6.1
6	*Journal of Medical Systems*	14	196	14	3.5
7	*Current Opinion in Anesthesiology*	12	118	9.83	2.3
8	*Anesthesiology*	9	685	76.11	9.1
9	*Computers in Biology and Medicine*	9	49	5.44	7.0
10	*Journal of Cardiothoracic and Vascular Anesthesia*	8	42	5.25	2.3

### Literature co-citation analysis

A visualization analysis was conducted on 114 papers that had been co-cited more than 8 times (Supplementary Fig. 1). The top 10 papers in terms of co-citations were listed in Table 2. Among them, five papers discussed the application of AI in the prediction (Connor 2019; Hatib et al. 2018; Hashimoto et al. 2020; Kendale et al. 2018; Wijnberge et al. 2020) of hypotension, and others included the prediction of hypoxemia, monitoring of the DoA, and prediction of complications. AI has been extensively studied for the prediction of hypotension and is a hotspot and frontier in the field.

**Table 2:** The top 10 studies in terms of co-citations

Rank	Article title	Journal	Year	Co-cited
1	Machine-learning algorithm to predict hypotension based on high-fidelity arterial pressure waveform analysis	*Anesthesiology*	2018	47
2	Artificial intelligence in anesthesiology: current techniques, clinical applications, and limitations	*Anesthesiology*	2020	34
3	Supervised machine-learning predictive analytics for prediction of postinduction hypotension	*Anesthesiology*	2018	31
4	Effect of a machine learning-derived early warning system for intraoperative hypotension vs standard care on depth and duration of intraoperative hypotension during elective noncardiac surgery: the HYPE randomized clinical trial	*JAMA*	2020	29
5	Explainable machine-learning predictions for the prevention of hypoxemia during surgery	*Nat Biomed Eng*	2018	25
6	A primer for EEG signal processing in anesthesia	*Anesthesiology*	1998	24
7	Artificial intelligence and machine learning in anesthesiology	*Anesthesiology*	2019	22
8	Anesthesia awareness and the bispectral index	*N Engl J Med*	2008	21
9	The influence of method of administration and covariates on the pharmacokinetics of propofol in adult volunteers	*Anesthesiology*	1998	21
10	Relationship between intraoperative mean arterial pressure and clinical outcomes after noncardiac surgery: toward an empirical definition of hypotension	*Anesthesiology*	2013	20

### Keyword co-occurrence cluster analysis

**Fig. 6:**
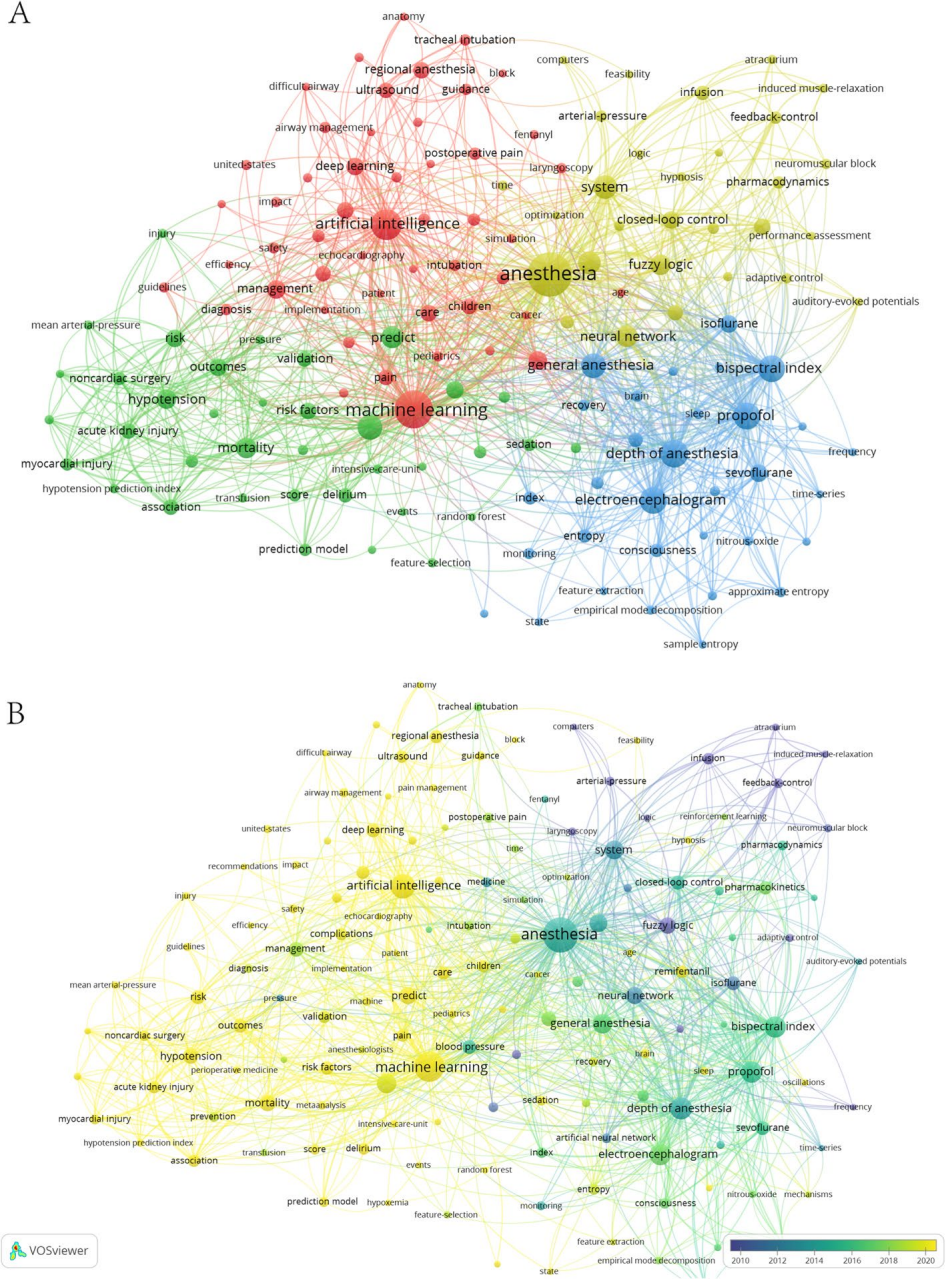
Co-occurrences of keywords in AI and anesthesiology identified by VOSviewer. **A** Network visualization: each node was a keyword, with the node size reflecting the number of times the keyword appeared. Connections between nodes indicate co-occurrence of keywords, and the thickness of the lines indicates the frequency of co-occurrences. **B** Overlay visualization: the average time of keyword appearances

The keywords, which serve as an overview of the core content of the paper, can be used to analyze the hotspots and frontiers. The keyword co-occurrence was visualized through VOSviewer. A total of 2221 keywords were identified, 148 of which appeared more than 5 times (Fig. 6A). The keywords were clustered into four categories based on research directions: Cluster 1 includes regional anesthesia, ultrasound, postoperative pain, and airway management. Cluster 2 includes hypotension, acute kidney injury, delirium, mortality, transfusion, hypoxemia, myocardial injury, predict, surgery, and algorithm. Cluster 3 includes depth of anesthesia, bispectral index, awareness, electroencephalogram, propofol, sevoflurane, and isoflurane. Cluster 4 includes closed-loop control, remifentanil, neuromuscular block, and propofol anesthesia. The overlay visualization (Fig. 6B) revealed the average year of appearance of keywords. The terms “regional anesthesia,” “ultrasound,” “pain,” “postoperative pain,” “airway management,” and “difficult airway” were identified 20, 17, 16, 10, 8, and 6 times, respectively. The occurrence of “mortality,” “hypotension,” “acute kidney injury,” “delirium,” “myocardial injury,” “transfusion,” and “hypoxemia” was observed to be 30, 28, 16, 15, 10, 6, and 5 times, respectively. The average time of appearance is approximately 2020, suggesting that these areas are the current hotspots and frontiers of AI in anesthesiology. The terms “depth of anesthesia,” “electroencephalogram,” “propofol,” “bispectral index,” “isoflurane,” “sevoflurane,” and “awareness” appeared 58, 54, 54, 53, 22, 21, and 15 times, respectively. The average time is about 2015, indicating that the research of AI in DoA monitoring started in an early stage, with considerable studies. The keywords “closed-loop control,” “remifentanil,” “neuromuscular block,” and “propofol anesthesia” appeared 22, 17, 7, and 6 times respectively. Except for remifentanil, which appeared around 2020, other drugs infusion appeared around 2010, suggesting that the overall research on drug infusion systems started earlier. Table 3 presents the top 20 keywords by frequency of appearance as identified by VOSviewer.

**Table 3:** The top 20 burst words in AI and anesthesiology presented by VOSviewer

Rank	Key word	Occurrences
1	Anesthesia	167
2	Machine learning	120
3	Artificial intelligence	74
4	Depth of anesthesia	58
5	Electroencephalogram	54
6	Propofol	54
7	Bispectral index	53
8	Surgery	47
9	General anesthesia	46
10	System	44
11	Models	39
12	Neural network	35
13	Predict	34
14	Fuzzy logic	30
15	Mortality	30
16	Hypotension	28
17	Classification	26
18	Management	24
19	Outcomes	23
20	Closed-loop control	22

**Fig. 7:**
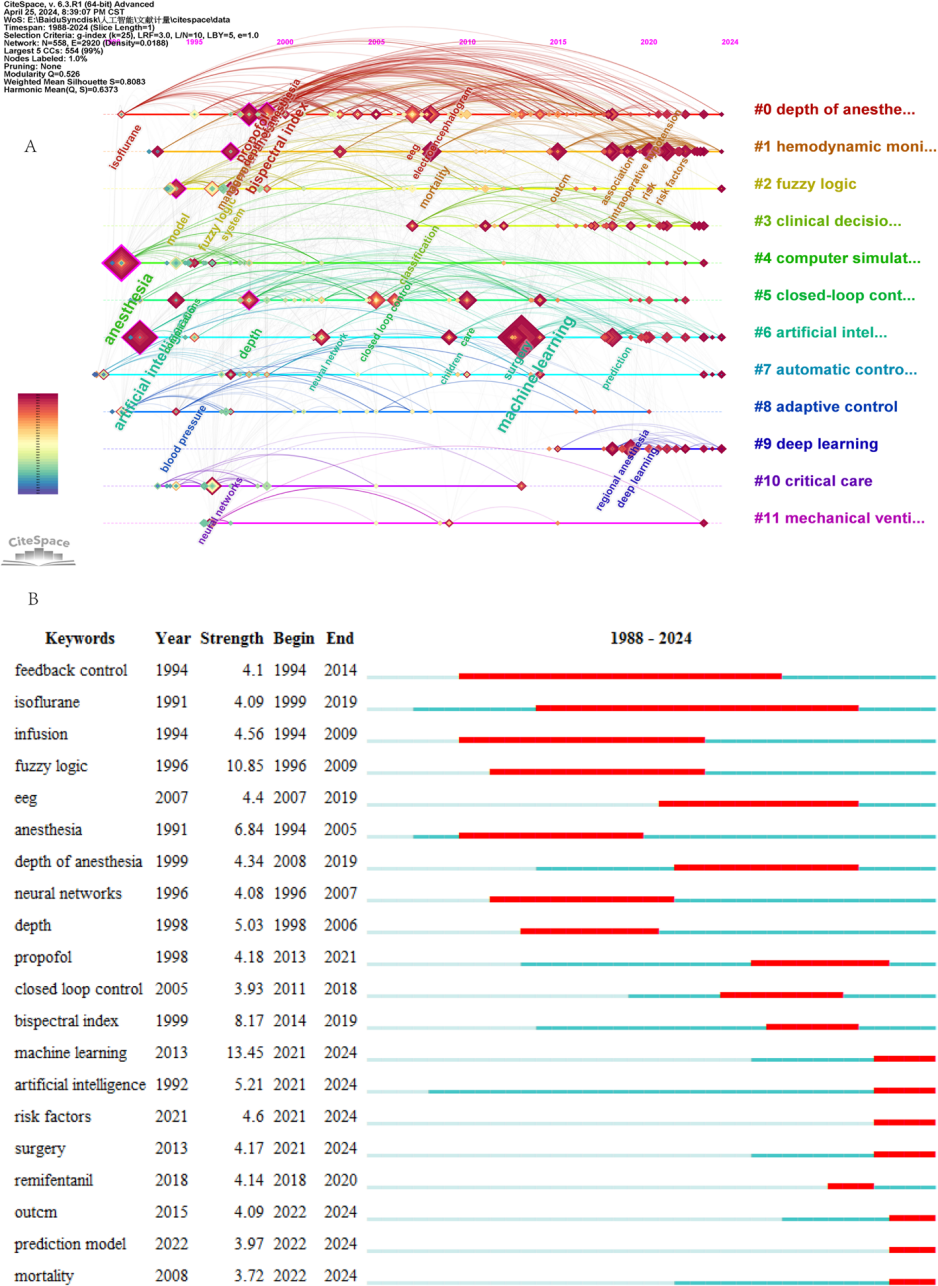
Co-occurrences of keywords in AI and anesthesiology identified by CiteSpace. **A** Keyword clustering timeline visualization. **B** The top 20 burst words

The keyword clustering timeline visualization (Fig. 7A), which was generated by CiteSpace, revealed that a significant number of keywords were present in the “depth of anesthesia” cluster. The keyword that appeared earliest was “isoflurane,” whereas the most frequently studied were “propofol,” “electroencephalogram,” and “bispectral index,” while the most recent keyword was “automated propofol sedation.” The “artificial intelligence” cluster appeared in anesthesiology around the 1990s, yet it was not the subject of extensive research at that time. It was only around the year 2012 that the application of “machine learning” began to gain traction in anesthesiology, with an increasing focus on the prediction of adverse events and the management of difficult airways, and the utilization of the most recent AI was highlighted for “operating room management.” The keywords in the “deep learning” cluster were more recent, with their emergence beginning approximately 2015. By approximately 2018, deep learning was applied in “regional anesthesia,” and the latest application was in “ultrasound.”

Burst analysis could reflect the research hotspots and frontiers of a certain period to a certain extent. The burst feature of CiteSpace was used to display burst words in AI and anesthesiology (Fig. 7B). The burst words “artificial intelligence,” “machine learning,” “prediction model,” and “risk factors” had a late emergence, which indicated that they were the frontiers in AI and anesthesiology in recent years.

## Discussion

**Fig. 8:**
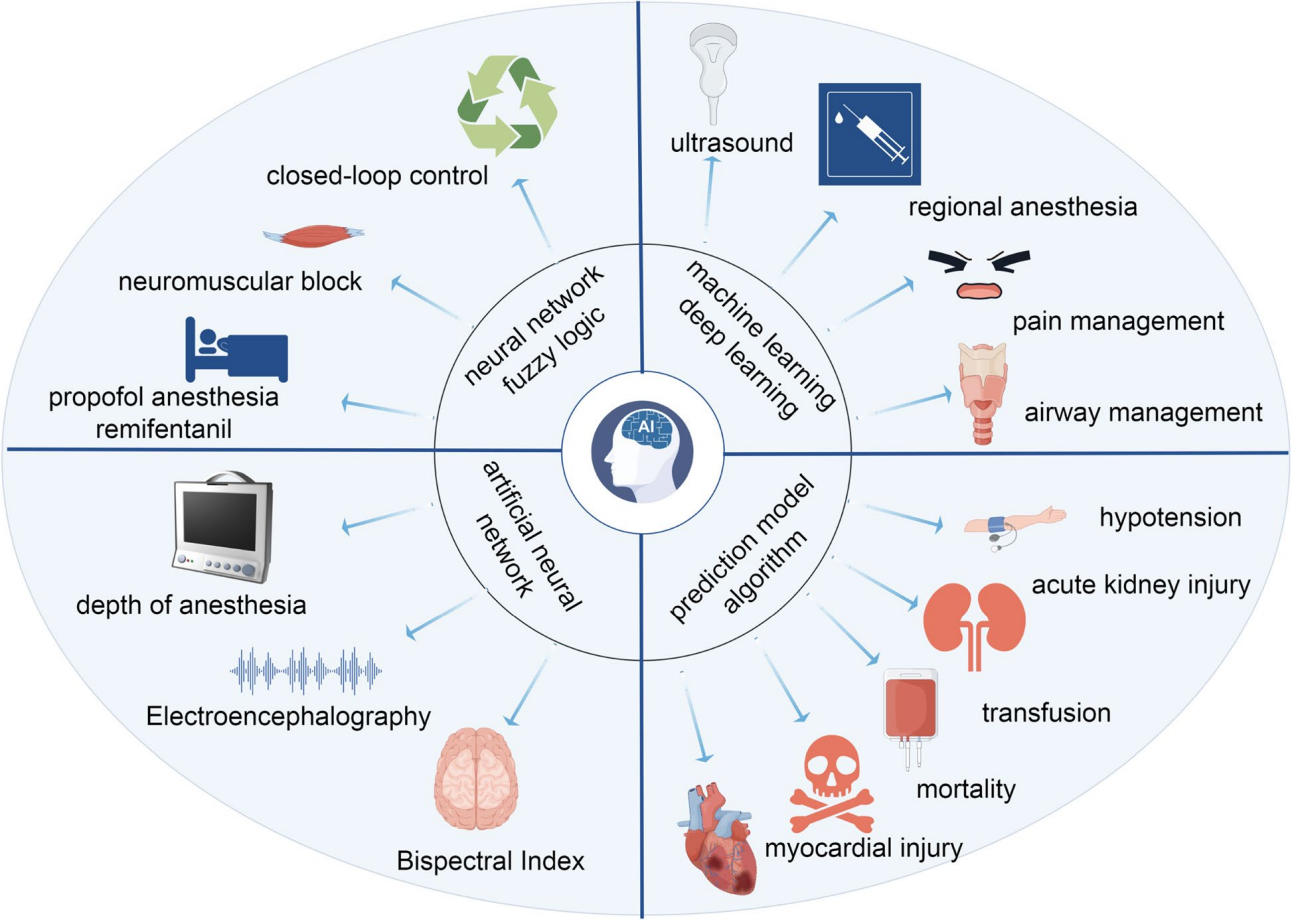
Application of AI in anesthesiology

In our study, a bibliometric analysis was used to ascertain the current status of AI in anesthesiology, including publication output, leading countries, preferred journals, top institutions, and clustered keywords. In summary, 491 papers from 48 countries, 715 institutions, and 218 journals were included in this article. The rapid increase number of papers since 2018 suggests that the study of AI in anesthesiology is attracting increasing interest. In terms of co-citation of literature, a significant amount of research was observed for the prediction of hypotension, marking it as a hot frontier issue. Through the keyword co-occurrence cluster analysis, the keywords could be categorized into four distinct clusters, including machine learning for ultrasound-guided regional anesthesia, prediction model, artificial neuronal network of DoA, and close-loop control of anesthesia (Fig. 8).

### Hotspots and frontiers of AI in anesthesiology

#### Research on AI in ultrasound-guided regional anesthesia

AI has been shown to have advantages in image recognition. Current research focuses on the use of deep neural networks for the identification of anatomical structures and needle positioning (Yang et al. 2022) (Mwikirize et al. 2021). New type of intelligent ultrasound device, such as the ScanNav Anatomy Peripheral Nerve Block, has already been proved by US FDA to help anesthetists and other health professionals identify anatomical structures in ultrasound images in 2022 (Bowness et al. 2022). The device uses deep learning technology to create color overlays of key anatomical structures on live ultrasound images. The device was first reported by Bowness and his team in 2022. The author explored its utility, which showed that ScanNav had potential to support nonexperts in training and clinical practice and experts in teaching ultrasound-guided regional anesthesia. Furthermore, the same team published two more studies in 2023. One study compared the accuracy of key anatomical structures of 720 videos acquired with or without the device (Bowness et al. 2023a), and the other evaluated ultrasound scanning performance by nonexperts (Bowness et al. 2023b). The first study found that the false-negative rate and false-positive rate of the device were 3.0% and 3.5%, respectively, and the risk of unwanted needle trauma and block failure were also judged to be reduced. The second study showed that use of AI device was associated with improved ultrasound image acquisition and interpretation for nonexperts. Nerveblox is another AI-driven decision support solution to help anesthesiologists practice peripheral nerve block faster, which could capture the real-time images from any ultrasonography device and label key anatomical landmarks on the images (Gungor et al. 2021). However, Nerveblox has not yet been approved for use on patients in the USA. Both Nerveblox and ScanNav Anatomy Peripheral Nerve Block could support the identification of some superficial peripheral nerves: interscalene/supraclavicular/infraclavicular brachial plexus, femoral nerve, saphenous nerve, popliteal sciatic nerve, erector spinae plane, and rectus sheath. Artificial neural network and machine learning have been used to automatically identify paravertebral or epidural anatomical structures and assist needle positioning, but they have not yet been applied to clinical practice (Zhao et al. 2023) (Pesteie et al. 2018). Further studies are necessary for AI to identify anatomy related to deep block such as subgluteal sciatic nerve block and improve the ability in visualizing the needle tip when the angle of penetration is acute (Viderman, et al. 2022). AI in ultrasound-guided regional anesthesia is still in its infancy and has a long way to go before it is widely used.

#### Research on AI in postoperative pain management

Currently, the assessment of pain is predominantly based on subjective descriptions or vital signs, without objective indicators, which may result in a degree of bias. AI is currently being explored to identify objective indicators of pain. During general anesthesia, the analgesia nociception index is used to assess pain levels, guiding the administration of opioids (Gonzalez-Cava et al. 2020). For the assessment of postoperative pain, researchers developed a novel analgesic index, namely spectrogram–convolutional neural network index, by combining photoplethysmogram spectrograms and a convolutional neural network (Choi et al. 2021). For the assessment of postoperative pain in children after cardiac surgery, studies had proposed using machine learning to understand pain behavioral responses based on facial expressions, body or head movements, and voice signal analysis (Pollak, et al. 2019). Preoperatively, AI was used to select patients who might benefit from preoperative pain consultation (Tighe, et al. 2012). In addition to pain assessment, AI can collect patients’ vital signs and pain data and predict the demand for analgesics after surgery through machine learning and deep learning analysis, making real-time adjustments and truly achieving dynamic, personalized preventive analgesia (Wang et al. 2020). In summary, AI may be used for pain assessment before, during, and after surgery and guiding treatment of postoperative pain. This might provide a closed loop of perioperative pain control.

#### Research on AI in airway management

Research on AI in airway management primarily focus on identifying airway structures, predicting difficult airways, and predicting the size and depth of tracheal tube intubation (Kim et al. 2023). Convolutional neural networks can recognize the vocal cords and tracheal rings in real time during video laryngoscopy (Matava et al. 2020). Deep learning aids in the development of automatic navigation systems to guide nasal tracheal intubation (Deng 2023). Deep learning was used to link patient facial images to the actual difficulty of intubation to establish a model for identifying difficult intubations (Hayasaka et al. 2021; Tavolara et al. 2021; Connor and Segal 2011). The accuracy of automated Mallampati classification was improved by deep convolutional neural networks, which can help clinicians objectively and accurately classify difficult airways (Zhang et al. 2022). Currently, many models have been established, but there is still no rapid and accurate device for identifying difficult airways in clinical practice. It remains to be verified whether the same model can be applied between different races.

#### Research on AI in prediction model

AI was utilized in the establishment of prediction models, including the prediction of hypotension, acute kidney injury, delirium, myocardial injury, transfusion, hypoxemia, and mortality (Fritz et al. 2019; Chen et al. 2022; Bishara et al. 2022). With these advanced predictive models, healthcare professionals can identify risks more accurately, optimize treatment plans, and improve patient outcomes and quality of life. The first to be applied in the operating room was the hypotension prediction index, a machine learning algorithm that predicts hypotensive events through high-fidelity analysis of pulse-wave contours (Hatib et al. 2018). Studies have validated the hypotension prediction index and found that it could reduce intraoperative hypotension compared with standard care (Wijnberge et al. 2020). However, the algorithm of hypotension prediction index was considered as a “black box,” which cannot indicate the causes of hypotension, necessitating further standardization and improvement, which needed further standardization and improvement (Ven et al. 2021).

#### Research on AI in DoA monitoring

With the advancement of AI, the processing of complex electroencephalogram (EEG) signals has become easier, and the number of related studies is increasing (Chen, et al. 2022), which has injected new vitality into the monitoring of the DoA. For example, a new index for accurately predicting DoA based on EEG signals, known as EEGMAC, was proposed by combining deep learning techniques. This index was applicable to patients under volatile and intravenous anesthesia and was found to be more effective than the bispectral index (Park et al. 2020). In addition, several studies have combined AI with auditory-evoked potentials to monitor DoA (Elkfafi, et al. 1998; Lu et al. 1999; Allen and Smith 2001). However, further validation is needed to determine which indicator is more sensitive to depth of anesthesia. With the development of AI, DoA monitoring has become more diversified and popularized, which will inevitably lead to new developments in closed-loop drug infusion for anesthetics.

#### Research on AI in perioperative drug infusion

With the development of technology, infusion pumps have evolved from constant-rate infusion to target-controlled infusion and closed-loop infusion systems. A comprehensive closed-loop infusion system during surgery requires an accurate and comprehensive monitoring and feedback system, including the DoA (sedation, analgesia, muscle relaxation), hemodynamics (SpO2, ECG, blood pressure), respiratory dynamics, and body temperature, among others (Ghita et al. 2020). Current research on closed-loop systems is limited to smaller scopes, such as closed-loop fluid and vasoactive drug management, DoA, muscle relaxation, and blood glucose control (Coeckelenbergh, et al. 2050; Fields et al. 2008). There is still a significant journey ahead to achieve full closed-loop management throughout the entire surgical procedure. Of course, anesthesiologist continue to be the most critical role in intraoperative management. Closed-loop infusion is merely a tool, with the ultimate decision-making authority resting in the hands of the anesthesiologist.

### Limitations and concerns of AI

Researches on AI in the field of anesthesiology is actively ongoing, but its limitations are also becoming increasingly apparent. Firstly, AI analyzes results from existing large volumes of data, which relies on the authenticity and accuracy of the data. However, the accuracy of the data is difficult to assess, necessitating the establishment of regulatory bodies. Obtaining large datasets is relatively challenging, requiring significant human resources, financial investment, and collaboration among multiple institutions (Kelly et al. 2019). Currently, the datasets used to train AI often come from specific populations and may only be applicable to certain groups of people (Maheshwari, et al. 2023). Secondly, the algorithms of deep learning are opaque, and it is not possible to analyze the reasons leading to the results, which leads to skepticism among physicians regarding the outcomes derived from deep learning (Hashimoto et al. 2020). Additionally, there is concern that advancements in AI might lead to the deterioration of clinical doctors’ skills (Maheshwari, et al. 2023). Lastly, the issue of accountability when AI-generated conclusions adversely affect patients is a matter that needs consideration (Solanki, et al. 2021). Clinical anesthesia deals with patients who exhibit individual differences, and the formulation of medical decisions is intricate and complex, unlike chess, which has clear rules. Often, decisions must be based on a comprehensive analysis of the clinical situation. Therefore, the focus of future AI research should not be to replace the judgment or skills of clinical doctors but to optimize their methods and increase the transparency of algorithms (Hashimoto et al. 2020).

### Limitations

This study was the first to use bibliometric visualization to analyze research on AI in anesthesiology. However, this study had several limitations. First, the search was limited to the Web of Science and did not include relevant literature from other databases, which might have resulted in an incomplete search. Second, this study included only English-language literature, which might have led to biased results. Third, the use of different names for the same institution at different times can lead to inaccuracies in the number of documents. Fourth, this paper only uses two software, VOSviewer and CiteSpace, and does not use others, such as the bibliometric online platform and R software, which reduces multiple verification. Fifth, this paper gives a brief overview of the research of AI in anesthesiology, in order to understand the application of AI in specific fields (such as airway management, pain management), and more in-depth literature reading is needed.

## Conclusion

The use of AI in various subfields of anesthesiology, including ultrasound-guided regional anesthesia, postoperative pain management, airway management, hypotension prediction, DoA monitoring, and intraoperative anesthetic drug infusion, has been studied; these fields are hot topics and frontiers. AI has demonstrated significant advantages in image recognition, data analysis, and the construction of predictive models, helping to improve the safety, accuracy, and efficiency of anesthesia. Despite the broad prospects for the application of AI in anesthesiology, there are also some challenges, such as the transparency of algorithms and ethical issues. Future research needs to further explore and refine these technologies while addressing the relevant regulatory and ethical issues to achieve optimal application of AI in anesthesiology.

## Supplementary Information

Supplementary Material 1: Supplementary Figure 1. Literature co-citation. The co-citation relationships among 114 papers that were co-cited more than eight times. Each node represents a paper, and the lines represent instances where two papers were co-cited. Supplementary Table 1. The top 10 institutions in terms of the number of publications. Supplementary Table 2. The top 5 authors in terms of the number of publications.

## Data Availability

No datasets were generated or analysed during the current study.

## Abbreviations

AI: Artificial intelligence
EEG: Electroencephalography
DoA: Depth of anesthesia

## References

[CR1] Allen R, Smith D. Neuro-fuzzy closed-loop control of depth of anaesthesia. Artif Intell Med. 2001;21(1–3):185–91. 11154884 10.1016/s0933-3657(00)00084-1

[CR2] Bishara A, et al. Postoperative delirium prediction using machine learning models and preoperative electronic health record data. BMC Anesthesiol. 2022;22(1):8. PMID: 34979919. 34979919 10.1186/s12871-021-01543-y PMC8722098

[CR3] Bowness JS, et al. Exploring the utility of assistive artificial intelligence for ultrasound scanning in regional anesthesia. Reg Anesth Pain Med. 2022;47(6):375–9. PMID: 35091395. 35091395 10.1136/rapm-2021-103368 PMC9046753

[CR4] Bowness JS, et al. Assistive artificial intelligence for ultrasound image interpretation in regional anaesthesia: an external validation study. Br J Anaesth. 2023a;130(2):217–25. PMID: 35987706. 35987706 10.1016/j.bja.2022.06.031 PMC9900723

[CR5] Bowness JS, et al. Evaluation of the impact of assistive artificial intelligence on ultrasound scanning for regional anaesthesia. Br J Anaesth. 2023b;130(2):226–33. PMID: 36088136. 36088136 10.1016/j.bja.2022.07.049 PMC9900732

[CR6] Chen Q, et al. Application of machine learning algorithms to predict acute kidney injury in elderly orthopedic postoperative patients. Clin Interv Aging. 2022;17:317–30. PMID: 35386749. 35386749 10.2147/CIA.S349978 PMC8979591

[CR7] Chen XL, et al. Global research on artificial intelligence-enhanced human electroencephalogram analysis. Neural Comput Appl. 2022;34(14):11295–333. PMID: WOS:000605918300007.

[CR8] Choi BM, et al. Novel analgesic index for postoperative pain assessment based on a photoplethysmographic spectrogram and convolutional neural network: observational study. J Med Internet Res. 2021;23(2):e23920. PMID: 33533723. 33533723 10.2196/23920 PMC7889419

[CR9] Coeckelenbergh S, et al. Perioperative fluid and vasopressor therapy in 2050: from experimental medicine to personalization through automation. Anesthesia Analgesia. 2024;138(2):284–94. 38215708 10.1213/ANE.0000000000006672

[CR10] Connor CW. Artificial intelligence and machine learning in anesthesiology. Anesthesiology. 2019;131(6):1346–59. PMID: WOS:000496457600024. 30973516 10.1097/ALN.0000000000002694 PMC6778496

[CR11] Connor CW, Segal S. Accurate classification of difficult intubation by computerized facial analysis. Anesth Analg. 2011;112(1):84–93. PMID: 21081769. 21081769 10.1213/ANE.0b013e31820098d6

[CR12] Deng Z, et al. Automatic endoscopic navigation based on attention-based network for nasotracheal Intubation. Biomed Signal Process Control. 2023;86:105035.

[CR13] Elkfafi M, et al. Fuzzy logic for auditory evoked response monitoring and control of depth of anaesthesia. Fuzzy Sets Syst. 1998;100(1–3):29–43. PMID: WOS:000077238100003.

[CR14] Epstein BS. ASA adopts standards for the practice of anesthesiology. Arch Surg. 1987;122(10):1215–6. 3662805 10.1001/archsurg.1987.01400220125027

[CR15] Fields AM, Fields KM, Cannon JW. Closed-loop systems for drug delivery. Curr Opin Anaesthesiol. 2008;21(4):446–51. 18660650 10.1097/ACO.0b013e3283007ecc

[CR16] Fritz BA, et al. Deep-learning model for predicting 30-day postoperative mortality. Br J Anaesth. 2019;123(5):688–95. PMID: 31558311. 31558311 10.1016/j.bja.2019.07.025 PMC6993109

[CR17] Ghita M, et al. Closed-loop control of anesthesia: survey on actual trends, challenges and perspectives. IEEE Access. 2020;8:206264–79.

[CR18] Gonzalez-Cava JM, et al. Machine learning based method for the evaluation of the analgesia nociception index in the assessment of general anesthesia. Comput Biol Med. 2020;118:103645. PMID: 32174322. 32174322 10.1016/j.compbiomed.2020.103645

[CR19] Gungor I, et al. A real-time anatomy identification via tool based on artificial intelligence for ultrasound-guided peripheral nerve block procedures: an accuracy study. J Anesth. 2021;35(4):591–4. PMID: 34008072. 34008072 10.1007/s00540-021-02947-3 PMC8131172

[CR20] Hashimoto DA, et al. Artificial intelligence in anesthesiology: current techniques, clinical applications, and limitations. Anesthesiology. 2020;132(2):379–94. PMID: 31939856. 31939856 10.1097/ALN.0000000000002960 PMC7643051

[CR21] Hatib F, et al. Machine-learning algorithm to predict hypotension based on high-fidelity arterial pressure waveform analysis. Anesthesiology. 2018;129(4):663–74. PMID: 29894315. 29894315 10.1097/ALN.0000000000002300

[CR22] Hayasaka T, et al. Creation of an artificial intelligence model for intubation difficulty classification by deep learning (convolutional neural network) using face images: an observational study. J Intensive Care. 2021;9(1):38. PMID: 33952341. 33952341 10.1186/s40560-021-00551-x PMC8101256

[CR23] John H, Eichhorn M, et al. Standards for patient monitoring during anesthesia at harvard medical school. JAMA. 1986;256(8):1017–20. 3735628

[CR24] Kelly CJ, et al. Key challenges for delivering clinical impact with artificial intelligence. BMC Med. 2019;17(1):195. PMID: 31665002. 31665002 10.1186/s12916-019-1426-2 PMC6821018

[CR25] Kendale S, et al. Supervised machine-learning predictive analytics for prediction of postinduction hypotension. Anesthesiology. 2018;129(4):675–88. PMID: 30074930. 30074930 10.1097/ALN.0000000000002374

[CR26] Kim H, et al. Predicting optimal endotracheal tube size and depth in pediatric patients using demographic data and machine learning techniques. Korean J Anesthesiol. 2023;76(6):540–9. PMID: 37750295. 37750295 10.4097/kja.23501 PMC10718635

[CR27] Lee J, et al. Comparative analysis on machine learning and deep learning to predict post-induction hypotension. Sensors (Basel). 2020;20(16):4575. PMID: 32824073. 32824073 10.3390/s20164575 PMC7472016

[CR28] Lonsdale H, et al. The perioperative human digital twin. Anesth Analg. 2022;134(4):885–92. PMID: 35299215. 35299215 10.1213/ANE.0000000000005916

[CR29] Lu, J.W.H.Y.Y., A. Nayak, R.J. Roy, *Depth of anesthesia estimation and control.* IEEE Trans Biomed Eng, 1999. 46(1). 10.1109/10.736759 9919828

[CR30] Maheshwari K, et al. Artificial intelligence for perioperative medicine: perioperative intelligence. Anesthesia Analgesia. 2023;136(4):637–45. PMID: WOS:000970613800003. 35203086 10.1213/ANE.0000000000005952

[CR31] Matava C, et al. A convolutional neural network for real time classification, identification, and labelling of vocal cord and tracheal using laryngoscopy and bronchoscopy video. J Med Syst. 2020;44(2):44. PMID: 31897740. 31897740 10.1007/s10916-019-1481-4

[CR32] Moon JS, Cannesson M. A century of technology in anesthesia & analgesia. Anesth Analg. 2022;135(2S Suppl 1):S48–61. PMID: 35839833. 35839833 10.1213/ANE.0000000000006027 PMC9298489

[CR33] Mwikirize C, et al. Time-aware deep neural networks for needle tip localization in 2D ultrasound. Int J Comput Assist Radiol Surg. 2021;16(5):819–27. 33840037 10.1007/s11548-021-02361-w

[CR34] Ninkov A, Frank JR, Maggio LA. Bibliometrics: methods for studying academic publishing. Perspect Med Educ. 2022;11(3):173–6. PMID: 34914027. 34914027 10.1007/s40037-021-00695-4 PMC9240160

[CR35] Park Y, et al. A real-time depth of anesthesia monitoring system based on deep neural network with large EDO tolerant EEG analog front-end. IEEE Trans Biomed Circuits Syst. 2020;14(4):825–37. PMID: 32746339. 32746339 10.1109/TBCAS.2020.2998172

[CR36] Pesteie M, et al. Automatic localization of the needle target for ultrasound-guided epidural injections. IEEE Trans Med Imaging. 2018;37(1):81–92. PMID: 28809679. 28809679 10.1109/TMI.2017.2739110

[CR37] Pollak, U., et al., *Postoperative pain management in pediatric patients undergoing cardiac surgery: where are we heading?* J Intensive Care Med, 2019: p. 885066619871432. PMID: 31446831. 10.1177/0885066619871432 31446831

[CR38] Rampil IJ. A primer for EEG signal processing in anesthesia. Anesthesiology. 1998;89:980–1002. 9778016 10.1097/00000542-199810000-00023

[CR39] Solanki SL, et al. Artificial intelligence in perioperative management of major gastrointestinal surgeries. World J Gastroenterol. 2021;27(21):2758–70. PMID: WOS:000660807300005. 34135552 10.3748/wjg.v27.i21.2758 PMC8173379

[CR40] Tavolara TE, et al. Identification of difficult to intubate patients from frontal face images using an ensemble of deep learning models. Comput Biol Med. 2021;136:104737. PMID: 34391000. 34391000 10.1016/j.compbiomed.2021.104737 PMC8440461

[CR41] Tighe PJ, et al. Use of machine-learning classifiers to predict requests for preoperative acute pain service consultation. Pain Med. 2012;13(10):1347–57. 22958457 10.1111/j.1526-4637.2012.01477.x PMC4012229

[CR42] van der Ven WH, et al. One of the first validations of an artificial intelligence algorithm for clinical use: the impact on intraoperative hypotension prediction and clinical decision-making. Surgery. 2021;169(6):1300–3. PMID: 33309616. 33309616 10.1016/j.surg.2020.09.041

[CR43] Viderman D, et al. Artificial intelligence in ultrasound-guided regional anesthesia: a scoping review. Front Med. 2022;9:994805. PMID: WOS:000880851500001. 10.3389/fmed.2022.994805 PMC9640918 36388935

[CR44] Wang R, et al. From patient-controlled analgesia to artificial intelligence-assisted patient-controlled analgesia: practices and perspectives. Front Med (Lausanne). 2020;7:145. PMID: 32671076. 32671076 10.3389/fmed.2020.00145 PMC7326064

[CR45] Wijnberge M, et al. Effect of a machine learning-derived early warning system for intraoperative hypotension vs standard care on depth and duration of intraoperative hypotension during elective noncardiac surgery: the HYPE randomized clinical trial. JAMA. 2020;323(11):1052–60. PMID: 32065827. 32065827 10.1001/jama.2020.0592 PMC7078808

[CR46] Yang XY, et al. Artificial intelligence using deep neural network learning for automatic location of the interscalene brachial plexus in ultrasound images. Eur J Anaesthesiol. 2022;39(9):758–65. PMID: 35919026. 35919026 10.1097/EJA.0000000000001720

[CR47] Zhang F, et al. Critical element prediction of tracheal intubation difficulty: automatic Mallampati classification by jointly using handcrafted and attention-based deep features. Comput Biol Med. 2022;150:106182. PMID: 36242810. 36242810 10.1016/j.compbiomed.2022.106182

[CR48] Zhao Y, et al. Utility of artificial intelligence for real-time anatomical landmark identification in ultrasound-guided thoracic paravertebral block. J Digit Imaging. 2023;36(5):2051–9. PMID: 37291383. 37291383 10.1007/s10278-023-00851-8 PMC10501964

