# Supplementary material for: Artificial intelligence in anesthesiology: a bibliometric analysis
Source: Perioper Med (Lond). 2024 Dec 23;13:121. doi: 10.1186/s13741-024-00480-x (PMC11668081; doi:10.1186/s13741-024-00480-x)
Supplement: Supplementary file 1 — Supplementary Material 1: Supplementary Figure 1. Literature co-citation. The co-citation relationships among 114 papers that were co-cited more than eight times. Each node represents a paper, and the lines represent instances where two papers were co-cited. Supplementary Table 1. The top 10 institutions in terms of the number of publications. Supplementary Table 2. The top 5 authors in terms of the number of publications. [file 13741_2024_480_MOESM1_ESM.docx]

**Supplementary Table 1**. The top 10 institutions in terms of the number of publications.

| Rank | Institution | Publications | Total Citations | Average Citations | |
| --- | --- | --- | --- | --- | --- |
| 1 | Yuan Ze Univ | 20 | 404 | 20.20 |  |
| 2 | Natl Taiwan Univ | 18 | 403 | 22.39 |  |
| 3 | Brunel Univ London | 13 | 319 | 24.54 |  |
| 4 | Stanford Univ | 12 | 211 | 17.58 |  |
| 5 | Seoul Natl Univ | 11 | 152 | 13.82 |  |
| 6 | Univ Sheffield | 10 | 238 | 23.80 |  |
| 7 | Univ Toronto | 10 | 154 | 15.40 |  |
| 8 | Vanderbilt Univ | 9 | 49 | 4.90 |  |
| 9 | Nanjing Med Univ | 9 | 9 | 1.00 |  |
| 10 | Capital Med Univ | 8 | 10 | 1.25 |  |

**Supplementary Table 2**. The top 5 authors in terms of the number of publications.

| Rank | Author | Publications | Total Citations | Average Citations |
| --- | --- | --- | --- | --- |
| 1 | Shieh,Jiann-Shing | 18 | 386 | 21.44 |
| 2 | Fan, Shou-Zen | 16 | 347 | 21.69 |
| 3 | Linkens, DA | 13 | 322 | 24.77 |
| 4 | Abbod, Maysam F | 13 | 319 | 24.54 |
| 5 | Lee, Hyung-Chul | 8 | 111 | 13.88 |


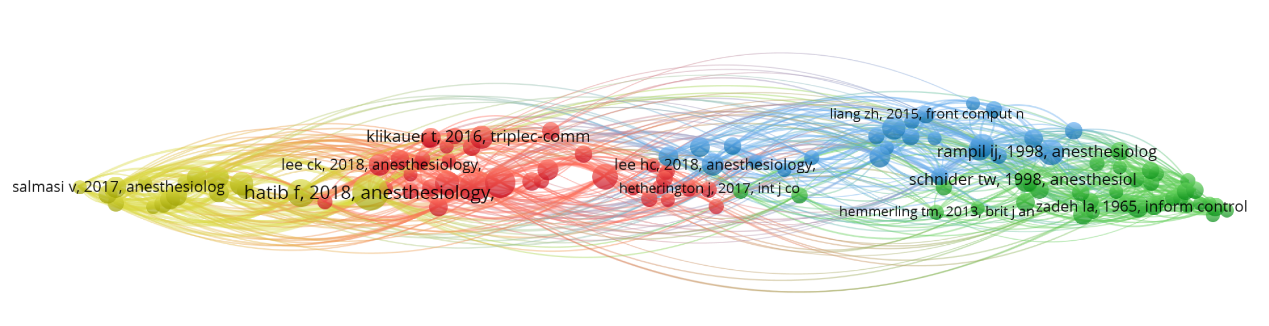


Supplementary Figure 1. Literature co-citation. The co-citation relationships among 114 papers that were co-cited more than eight times. Each node represents a paper, and the lines represent instances where two papers were co-cited.
